# Unmet clinical needs and burden in Angelman syndrome: a review of the literature

**DOI:** 10.1186/s13023-017-0716-z

**Published:** 2017-10-16

**Authors:** Anne C. Wheeler, Patricia Sacco, Raquel Cabo

**Affiliations:** 1 RTI International, 3040 Cornwallis Road, PO Box 12194, Research Triangle Park, NC 27709-2194 USA; 2 RTI Health Solutions, 200 Park Offices Drive, Research Triangle Park, NC 27709 USA; 3 Ovid Therapeutics Inc., 1460 Broadway, New York, NY 10036 USA

**Keywords:** Angleman syndrome, Burden, Unmet clinical needs, Clinical features, Treatments

## Abstract

**Background:**

Angelman syndrome (AS) is a rare disorder with a relatively well-defined phenotype. Despite this, very little is known regarding the unmet clinical needs and burden of this condition, especially with regard to some of the most prevalent clinical features—movement disorders, communication impairments, behavior, and sleep.

**Main text:**

A targeted literature review using electronic medical databases (e.g., PubMed) was conducted to identify recent studies focused on specific areas of the AS phenotype (motor, communication, behavior, sleep) as well as epidemiology, diagnostic processes, treatment, and burden. 142 articles were reviewed and summarized. Findings suggest significant impairment across the life span in all areas of function. While some issues may resolve as individuals get older (e.g., hyperactivity), others become worse (e.g., movement disorders, aggression, anxiety). There are no treatments focused on the underlying etiology, and the symptom-based therapies currently prescribed do not have much, if any, empirical support.

**Conclusions:**

The lack of standardized treatment protocols or approved therapies, combined with the severity of the condition, results in high unmet clinical needs in the areas of motor functioning, communication, behavior, and sleep for individuals with AS and their families.

## Background

Angelman syndrome (AS) is a rare neurodevelopmental disorder caused by lack of expression of the maternal ubiquitin-protein ligase E3A (*UBE3A*) gene in the brain [1, 2]. There are 4 known etiologies of AS responsible for the silencing of the *UBE3A* gene: deletion in chromosome 15q11-q13 (70% of cases), paternal uniparental disomy (UPD; 2% of cases), imprinting defect (3% of cases), and point mutation (10% of cases) [3]. There are 2 documented deletion types classified based on the proximal breakpoint (BP)—class I (BP1-BP3) and class II (BP2-BP3). Class I deletions are bigger, with implications for greater severity in phenotype [4–7]. Approximately 10% of individuals with a clinical phenotype of AS who undergo genetic testing do not have genetic confirmation of the syndrome. The prevalence of AS is generally estimated to be approximately 1:15,000 births, although the true prevalence is not well characterized.

As a result of missing *UBE3A* in the brain, individuals with AS have severe to profound intellectual disability (ID), lack of speech, difficulties with motor control and planning, significant sleep difficulties, seizures, and unique behavioral features [4, 8]. The AS clinical phenotype has been widely reviewed [4, 8–13]. A consensus report in 1995 [14] and updated in 2005 [9] lists features seen in 100% of individuals with AS (consistent) as well as features seen in 80% (frequent) and features seen in 20%–80% (associated). These consensus criteria are typically used as a basis for a clinical diagnosis, which is then confirmed through genetic testing. There is no evidence to suggest that individuals with AS experience a shorter-than-expected life span, and some symptoms may become more severe over time [4, 12, 15]. There are currently no AS-specific systematic treatment approaches; treatment and management is symptomatic with no therapy that addresses the underlying etiology.

The unmet clinical need and quality-of-life impact of associated features of AS have not been well described. The chronic and severe nature of these features of AS is thought to result in substantial burden for caregivers, although there has been little empirical documentation of the psychological, behavioral, and physiological effects on the daily lives and health of caregivers. The primary goal of this targeted review is to characterize the unmet clinical need of AS. We highlight literature on the 3 most common phenotypic characteristics (other than global developmental delay) from the “consistent” category of the clinical diagnostic criteria: movement disorders, speech and communication impairments, and unique behavioral characteristics. In addition, we review the recent literature on sleep disturbance, as this is one of the more significant contributors to poor health-related quality of life (HR-QOL) for individuals with AS and their caregivers. The secondary objective is to provide an overview of the epidemiology and burden of AS on individuals and their families and caregivers.

In making the clinical diagnosis of AS, seizures are included as a frequent feature. Seizure types that are typical of AS include atypical absence, myoclonic, and nonconvulsive status epilepticus [16]. Seizures that accompany AS are generally better understood, as compared with movement disorders, communication impairments, and behavioral characteristics, and there are medical therapies that have regulatory (e.g., Food and Drug Administration) approval to treat these specific types of seizures. For this reason, seizures have not been included as an area of focus in this literature review, although the authors recognize the clinical importance of effectively treating seizures in patients with AS.

## Main text

### Methods

Literature searches were performed using electronic medical databases on the phenotypic features and treatment options for AS in the following specific topic areas: motor impairments, communication challenges, behavior, and sleep disturbance. PubMed served as the primary database for the electronic literature search, with supplemental searches in Google Scholar. In addition, the bibliographies of existing literature reviews and key articles were reviewed to identify other relevant articles appropriate for inclusion. The results from the different searches were cross-referenced to identify and remove duplicates. The goal of the literature search strategy was to identify published articles for which the topic of interest was the primary focus, rather than all articles on the topic. Internet searches provided supplemental information, thus ensuring that interpretation of the identified articles was consistent with current knowledge. Table 1 provides the inclusion and exclusion criteria to screen the articles identified in the electronic searches. We excluded studies that were not published in the English language and those that did not report research results related to the key question. Articles focusing on motor, communication, behavior, and sleep, along with epidemiology, diagnostic processes, treatment, resource utilization, and caregiver burden were prioritized.

**Table 1:** Inclusion/Exclusion Criteria

Search Category	Inclusion/Exclusion Criteria
Study population	Individuals with AS, parents/caregivers of individuals with AS
Topic areas	Epidemiology of AS, natural history and description of the phenotype especially in the 4 targeted areas, diagnostic processes, caregiver burden and impact on family, treatment approaches and guidelines, resource utilization and associated health care costs
Dates	2000-present
Publication languages	English only

*AS* Angelman syndrome

The publications were screened at 2 levels. First, the titles and abstracts were reviewed to determine which studies were appropriate for inclusion. Second, once the abstracts were assessed for relevance, the full articles were retrieved for all the abstracts that met the relevance criteria. Finally, upon retrieval of the articles, each article was reviewed, abstracted, and summarized according to the area of focus (e.g., motor, communication, behavior, sleep).

## Results

**Fig. 1:**
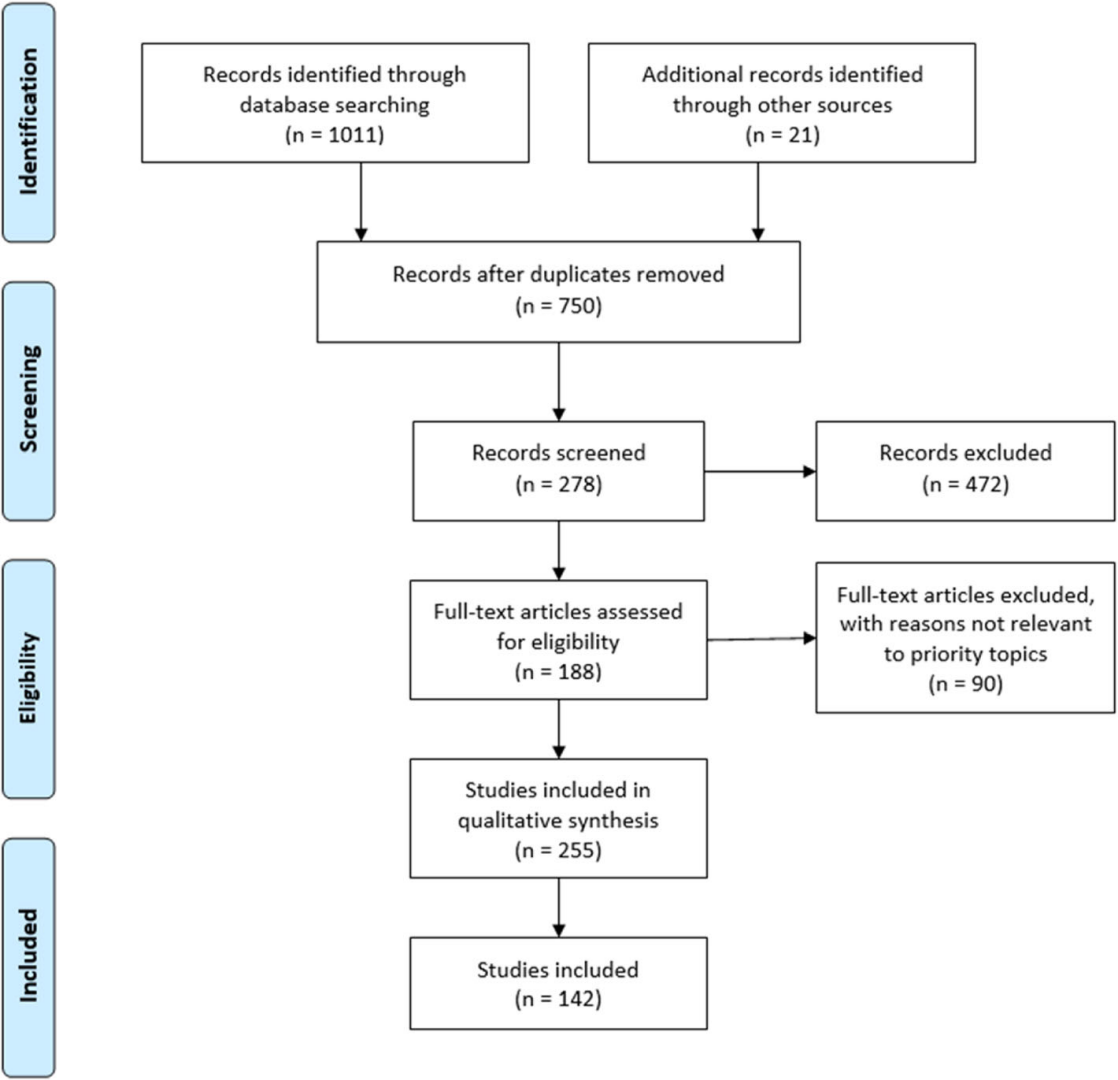
PRISMA 2009 Flow Diagram

The original search yielded 750 unique articles after removal of duplicates. After a review of titles and abstracts, 142 publications were included for full-text review. Figure 1 presents the PRISMA (i.e., Preferred Reporting Items for Systematic review and Meta-Analysis) flow chart, which details the number of publications identified in the literature search, the number of publications included and excluded at each phase, and the number of publications that met inclusion criteria.

### Epidemiology

AS is considered a rare disorder, with a commonly reported prevalence of approximately 1:15,000 births. However, reported estimates based on recent studies using different methodology have reported incidence rates between 1:10,000 and 1:62,000 [17–19]. Most of these recent studies examining prevalence have done so by exploring the number of cases of AS among populations of individuals with severe ID and/or epilepsy, then calculating incident rates relative to the larger population [18, 20, 21]. These studies, while valuable for providing estimates, provide only a sampling from the larger population, and therefore may not reflect the true incidence of the disorder. As a result, there is likely a number of undiagnosed or misdiagnosed individuals with AS. (See Table 2 for a summary of these findings.)

**Table 2:** Summary of prevalence studies

	Target	No. Positive/No. Tested	Prevalence Rates Found	Methodology	Country
Clayton-Smith, 1995 [20]	Not reported	Not reported	1:62,000	Medical record review from 1989 to 1992 (only those under age 29)	United Kingdom
Kyllerman, 1995 [115]	Individuals with epilepsy and intellectual disability	4/48,873	1:12,000	Genetic testing of all children with epilepsy ages 6–13 with intellectual disability	Sweden
Mertz et al., 2013 [18]	All identified patients compared with livebirth records	51/1,253,599	1:24,580	Review of records in the Danish National Patient Registry and the Danish Cytogenetic Central Registry from 1991 to 2009	Denmark
Oiglane-Shlik et al., 2006 [21]	All identified patients compared with livebirth records	7/3,650,266	1:52,181	Country-wide search for children with known Angelman syndrome or Prader-Willi syndrome born between 1984 and 2004	Estonia
Petersen et al., 1995 [17]	Individuals seen in a neuropediatric clinic	5/500,000	1:10,000	Individuals in neuropediatric clinic from 1983 to 1991	Denmark
Thomson, et al., 2006 [19]	All identified patients compared with livebirth records	26/1,050,000	1:40,000	Retrospective, quantitative review of Disability Services Commission files from 1953 to 2003	Australia

Additionally, approximately 10% of individuals with a clinical phenotype of AS do not have genetic confirmation of the syndrome [22]. These “Angelman-like” cases may be misdiagnoses of phenotypically similar conditions such as Phelan-McDermid, Christianson, Mowat-Wilson, Kleefstra, or Rett syndromes [22]. It is also possible that there is another mechanism for repression of *UBE3A* expression that has not yet been identified in these cases [4].

### Unmet clinical needs

#### Movement disorders

Movement disorders are almost universal in AS with nearly all individuals having some form of motor impairment. (See Table 3 for a summary of the natural history of movement disorders in AS.) The most common motor problems include spasticity, ataxia of gait (observed in the majority of ambulatory individuals), tremor, coactivation of muscles during locomotion, muscle weakness, and sensory-motor integration [23]. While the majority of children develop mostly normal tone in childhood, approximately 25% have persistent hypotonia and approximately 30% develop hypertonia [6]. An increased risk for scoliosis, especially in adulthood [8] also contributes to movement disorders. All of these motor problems contribute to gross, fine, or oral motor impairment, including the development of motor milestones. The onset of sitting is about 12 months, and walking typically occurs around 3 years of age [6]. Motor skills are a noted weakness relative to other developmental skills [24]. These movement disorders also contribute to delays in the development of cognitive and functional skills, which tend to plateau at 24 to 30 months [24].

**Table 3:** Natural history of select Angelman syndrome symptoms

	Infancy/Early Childhood	Middle Childhood/Adolescence	Adulthood	Current Standard of Care
Movement disorders	▪ Hypotonia/“floppy infant” observed in ~50% [6] ▪ Feeding issues/Poor suck in infancy [6, 8, 15, 16] ▪ All motor milestones delayed [24]	▪ Tone improves in most, but ~25% have persistent hypotonia and ~30% have hypertonia [6] ▪ Atypical or ataxic gait is common [10, 23, 25, 26] ▪ Coactivation of muscles, muscle weakness, and sensory-motor integration are also common [22] ▪ Developmental skills tend to plateau at 24–30 months [24] ▪ Oral motor challenges are also pervasive, with tongue thrusting, sucking and swallowing disorders, frequent drooling, excessive chewing, mouthing behaviors, and feeding difficulties reported in 20%–80% [8, 15, 16] ▪ Full toilet training obtained for about 30% of individuals with AS, although age of obtaining this milestone varies [8] ▪ NEM usually starts in adolescence [16, 28]	▪ Increase in scoliosis [8] ▪ Increases in tremor and NEM may result in loss of previous obtained skills [16, 28]	▪ Physical and occupational therapy [12] ▪ Physiotherapy [12] ▪ Bracing as needed [12] ▪ Increased activity and diet to prevent obesity [12]
Speech/Communication	▪ Less cooing and babbling in infancy [15] ▪ None or very little oral speech acquisition [35]	▪ No increases in verbal output [35] ▪ Development of gestures and other methods of communication [35, 36, 41, 42] ▪ Receptive language better than expressive [37, 45]	▪ No increases in verbal output [35] ▪ With improvement in attention span in adulthood, some increases in nonverbal communication [12] ▪ Communication challenges thought to contribute to aggression and anxiety [75]	▪ Augmentative and alternative communication systems [34, 44] ▪ Promotion and enhancement of natural gestures [46–48]
Behavior				
*Smiling/Laughing*	▪ Early, persistent social smile reported as early as 1–3 months of age [50]	▪ More socially appropriate smiling/laughing [53]	▪ Decline in duration of smiling and laughing [54–56]	▪ Behavior-based interventions such as applied behavior analysis or discrimination training [77, 80–82] ▪ Increasing communication outputs [80] ▪ Psychopharmological treatment, including anxiolytic/anti-depressants (e.g., buspirone, guanfacine, clonidine); SSRIs (e.g., fluoxetine, sertraline, citalopram); antipsychotics (e.g., risperidone, aripiprazole, olanzapine); antihypnotic/stimulant (e.g., atomoxetine, methylphenidate, dexmethamphetamine) [16, 75]
*Hyperactivity*	▪ Hyperactivity reported to occur in nearly all young children with AS [15, 60]	▪ Hyperactivity/excitability the most prominent and severe behavior reported [13, 60]	▪ Hyperactivity decreases in adulthood [12, 60]	
*Aggression/Irritability*		▪ Physical aggression has been reported in up to 73% of adolescents with AS [74]	▪ Physical aggression/irritability increase in adulthood [75]	
*Autism symptoms*		▪ Comorbid autism diagnosis reported in up to 80% [62–64] ▪ Those with AS and comorbid ASD score lower on measures of language, adaptive behavior, and cognition, and have a slower rate of growth over time than those with AS without ASD [63]		
*Anxiety*			▪ Anxiety increase in adulthood [75]	
*Other behaviors (*e.g.*, repetitive behavior, mouthing)*			▪ Self-injurious behaviors increase in adulthood [75]	
Sleep	▪ Sleep issues peak between 2 and 9 years of age [84, 86]	▪ Sleep improves for some but sleep problems continue for a significant percentage [12, 75, 85–87] ▪ The most common type of sleep problem is insomnia with 35%–60% of individuals with AS being described as having difficulty initiating sleep and/or maintaining sleep and reduced total sleep time [61, 85, 87, 88] ▪ Quality of sleep is also problematic, with reduced sleep efficiency found via sleep polygraph in children with AS compared with controls [88, 89]	▪ Sleep improves for some, but sleep problems continue for up to 72% [12, 75, 85–87]	▪ Melatonin [93–97] ▪ Improvement of sleep environment [86, 87, 100] ▪ Adjusting sleep-wake schedules [94, 100] ▪ Reinforcing bedtime routines and independent sleep initiation [97, 100] ▪ Seizure medications [99, 116]

*AS* Angelman syndrome, *ASD* autism spectrum disorder, *NEM* non-epileptic myoclonus, *SSRI* selective serotonin reuptake inhibitors

Approximately 10% of individuals with AS never walk [25]. For those who do, gait can vary significantly, with some more mildly affected individuals having only a mild toe-walk or “prancing gait” while others are more stiff or extremely shaky and jerky when walking [10]. Ataxic gait often occurs with upraised arms and/or hand flapping [25, 26]. Fine motor skills are also significantly impaired, often due in part to difficulties with motor planning, tonicity, and tremor [24]. Hyperkinetic movements of the trunk and limbs, jitteriness, or tremulousness may be present in infancy [23], and increases in tremor into adulthood are common [8] and likely exacerbated by seizure treatments [16]. Tremors are made worse with stress [27]. Many individuals with AS also experience frequent jerking of the limbs, known as non-epileptic myoclonus (NEM). NEM consists of myoclonic jerks, lasting seconds to several hours, which usually start in the hands and spread to upper and lower extremities, face, and the entire body [28]. NEM tends to start during puberty and increase through adulthood; by age 40, all individuals with AS have some NEM, with daily to monthly frequency [16, 28].

Some studies show differences in motor skills by genetic subtype. Individuals with deletion have higher incidence of being nonambulatory and have more swallowing disorders, hypotonia, and ataxia, than those with UPD [7, 29]. Children with UPD are more likely to walk earlier (about 30 months) compared with children with a deletion (about 54 months) [30].

Oral motor challenges are also pervasive, with tongue thrusting, sucking and swallowing disorders, frequent drooling, excessive chewing, mouthing behaviors, and feeding difficulties during infancy reported in 20% to 80% of individuals with AS [8, 15, 16].

### Treatments

There are very few studies examining efficacy of specific treatments for movement disorders in AS, and most of the studies that have been done have been case reports. For example, in a case study, levodopa was found to decrease tremor and other Parkinsonism symptoms in 2 adults with AS [31], and intensive physiotherapy was found to result in dramatic improvements scores in gross motor function in one young child with AS, with a nearly 60% increase in total scores on gross motor function over the course of 36 months [32]. In one small non–placebo-controlled study of minocycline, some minor improvements in fine motor skills were found in children with AS aged 4 to 12 [33]; however, these results were not conclusive to suggest minocycline could make a lasting impact. There have been no systematic studies to examine the extent to which these therapies may help the majority of individuals with AS.

Standard of care for movement disorders typically involves assessment and intervention by physical therapists and occupational therapists. Physiotherapy or bracing is often recommended for scoliosis and to improve movement, while increased activity, full range of joint movement, and monitoring of diets are encouraged to prevent obesity and maintain mobility [12]. Physiotherapy has also been suggested as an effective intervention for increasing mobility in adults with AS [12], although there are no good efficacy studies to suggest long-term benefit. (See Table 4 for a summary of treatment studies addressing movement disorders.)

**Table 4:** Recent treatment studies for Angelman syndrome

Study	Number Of Participants	Symptom Target	Design	Intervention/Treatment	Outcomes
Harbord, 2001 [31]	2 adults with AS	Tremor/Parkinsonism	Case study	Levodopa	Both returned to being independently ambulant following treatment
Kara et al., 2010 [32]	1 young child with AS	Gross motor	Case study	Physiotherapy	During 36 months, gross motor function measurement increased from 11.46% to 70.82%
Grieco et al., 2014 [33]	25 children with AS	Cognitive and behavioral symptoms of AS	Single-arm, open-label	Minocycline	Significant improvements in fine motor, communication, and self-direction
De Carlos Isla et al., 2015 [34]	1 child with AS	Communication	Case study	Hanen—*More Than Words*	Significant increases in spontaneous interactions, quantity and quality of communicative acts, and increased rate of language development
Calculator, 2002 [46]	9 children with AS	Communication	Feasibility study	Home-based training of parents on promoting enhanced natural gestures for communication	Parents found this method to be acceptable, effective, reasonable, and easy to learn and teach
Calculator and Sela, 2015 [47]	3 children with AS	Communication	Case study	Teachers used strategies in the classroom to promote enhanced natural gestures for communication	Some efficacy for all 3 participants; 2 out of 3 of the students showed rapid and spontaneous use of enhanced natural gestures
Calculator, 2016 [48]	18 children with AS	Communication	Quasi-experimental	Home-based training of parents on promoting enhanced natural gestures for communication	Most children met or exceeded their predetermined communication goals
Radstaake et al., 2013 [80]	3 children with AS	Behavior	Case study	Functional analysis and functional communication training	In all 3 children, challenging behaviors decreased (large effect for 1 and a medium effect for the other 2) as a result of functional communication training
Summers, 2012 [81]	8 children with AS (4 in treatment, 4 in intervention)	Adaptive behavior	Nonrandomized pre-test/post-test control group design	Applied behavior analysis	Positive trends all 4 children in the intervention group for fine motor and receptive language. Not statistically significant, but suggestive of possible benefit.
Heald et al., 2013 [82]	4 children with AS	Social approach	Case study	Discrimination training	All 4 children showed reduced rates of social approach in response to a stimulus, suggesting this approach could provide a method for teaching children to recognize when adults are available in order to reduce inappropriate social approach
Takaesu et al., 2012 [93]	6 children with AS and documented circadian rhythm sleep disorders	Sleep	Case study	Melatonin	4 out of 6 children showed improvements in sleep patterns over 3 months of melatonin treatment
Summers et al., 1992 [97]	1 child with AS	Sleep	Case Study	Diphenhydramine hydrochloride before bedtime, combined with behavioral treatment consisting of not allowing the child to sleep during the day, restricting access to fluids at night, and going to bed at a consistent hour every night.	Night sleep increased from 1.9 h to 8.3 h and was maintained at 45 day follow up.
Braam et al., 2008 [94]	8 children with AS	Sleep	Randomized placebo-controlled study	2 doses of melatonin relative to placebo	Melatonin significantly improved sleep onset, decreased sleep latency, increased total sleep time, and reduced the number of night wakings in all 4 treatment group participants compared with the 4 participants in the placebo group
Forrest et al., 2009 [99]	4 children with AS	Sleep	Case study	Corticosteroid therapy	In addition to improving seizures and epileptic spasms, this treatment also improved developmental progress (e.g., increased alertness and responsiveness, improved fine motor skills) and sleep in all 4 children
Allen et al., 2013 [100]	5 children with AS	Sleep	Case study—multiple baseline	Behavioral treatment targeting sleep environment, sleep-wake schedule, and parent-child interactions during sleep time	Independent sleep initiation was increased for all participants; statistically significant changes in disruptive bedtime behaviors and in sleep onset
Radstaake et al., 2014 [104]	7 individuals with AS	Toilet training	Case study—A-design	Prompts for voiding with reinforcement	All showed some improvements, but only 3 maintained positive results after 3–18 months

*AS* Angelman syndrome

#### Speech/communication impairments.

Most individuals with AS do not ever develop oral speech or more than a few vocalizations, even with therapy [34]. For the few that do develop some oral speech, it primarily consists of fewer than 2 words or word approximations [34, 35]. In adulthood, there does not appear to be a significant increase in word use or vocalizations, with reports of an average of 5 words for adults [36]. Speech impairments are more severe than would be expected based on developmental or cognitive level [13, 37]. Dyspraxia is believed to play a significant role. (See Table 3 for a summary of the natural history of speech impairments in AS.) Those individuals with significant seizures who are on anticonvulsant medication and/or have extreme hyperactivity often have more delayed communication [15, 38, 39]. Difficulties with communication are thought to contribute to frustration and increases in aggression and anxiety.

Speech impairment is clearly present by 2 to 3 years of age, but there are signs of impairment earlier, with reports of infants with AS crying less often and engaging in less cooing and babbling [15]. Prelinguistic behaviors such as pointing, reaching, looking at, and giving objects have been observed as communicative gestures [40]. Several studies have found that nearly half of children with AS are able to produce meaningful, symbolic gestural communication to express a feeling or idea (e.g., signing “all done”) [35, 36, 41, 42]. By adulthood, most are able to convey needs and wants through multiple communication modalities [8, 42]. Communication attempts are most often to request or reject, with very few individuals with AS ever using communicative gestures to label objects or to imitate [38, 43]. Hand gestures, such as those used in sign language, are less likely used than whole hand, limb, or body gestures due to fine motor planning impairments [42]. Nonverbal communication skills, including gestures, have been found to improve in some individuals during adulthood, potentially in relation to an improvement in attention span [12]. Natural, self-developed gestures, nonspeech vocalizations, and physical manipulation were cited by parents as the most important communicative acts, more so than use of electronic communication aids [44].

Although both expressive and receptive language are significantly impaired in individuals with AS, several studies note receptive skills being significantly stronger than expressive skills [37, 42, 45]. This suggests that, while individuals are not able to verbally express themselves, they are able to understand at a higher developmental level verbal language directed toward them by others.

### Treatment

One nonplacebo, single-arm trial of minocycline found an improvement in auditory comprehension (but not expressive communication) in children ages 4 to 12 years diagnosed with AS [33]. However, the vast majority of communication interventions for children with AS have focused on augmentative and alternative communication systems [44]. Use of picture symbols, objects, iPads, and other electronic devices have been used to promote communication in this population [34, 44]. Parents have also been taught ways of building on natural motor movements of individuals with AS to promote and enhance natural gestures for communication [46–48]. Most studies exploring the efficacy of these interventions have found positive results; however, many of them require intensive time and effort to implement. Parents have noted that the burden of teaching these skills is more burdensome than the limits of having their child communicate in whatever unique way their child conveys their needs [49]. (See Table 4 for a summary of treatment studies addressing communication.)

#### Behavioral characteristics

Table 3 provides a summary of the natural history of select AS symptoms, including behavioral characteristics. Individuals with AS are described as having a unique behavioral phenotype that includes frequent laughing, smiling and excitability. While these characteristics are considered a “consistent” feature in the clinical diagnostic features, the literature suggests that the behavioral profile of individuals with AS is much more complex and variable, as is described in the following sections.

### Smiling/laughing

Excessive smiling and frequent, often inappropriate, laughing are hallmark behavioral features in AS, with up to 77% of individuals with AS reported to engage in frequent laughing, smiling, or happy demeanor [50, 51]. Individuals with AS have been observed to smile more in all contexts than those with other ID conditions [52]. The frequency and nature of smiling changes as the individual with AS ages. A persistent social smile has been reported to develop earlier than in typically developing infants, beginning as early as 1 to 3 months of age [50]. Socially appropriate smiling/laughing may develop a bit later in childhood [53] and then there is a reported decline in the duration of smiling and laughing [54] and the capacity for social interactions to evoke smiling and laughing in adolescence and adulthood [55, 56]. More severely cognitively impaired individuals are less likely to have an apparent happy demeanor [50]. However, adaptive behaviors were not correlated with smiling or laughing behaviors [57].

Although smiling/laughing does occur more in social settings, it is not always socially/contextually appropriate [54, 57]. This has led to a debate in the field as to whether these behaviors are considered a motor-expressive event triggered by a nonspecific stimulus [29] such as specific sounds (e.g., tuning fork) [58] or are socially driven [54, 57]. The finding in one study demonstrating increased social approach toward mothers when the mother was looking at them [59] suggests an important role (and potential burden) for caregivers in promoting socially appropriate communication and learning.

### Hyperactivity/excitability

Hyperactivity is one of the most frequent and severe behaviors reported for individuals aged 6 to 21 with AS [13, 60] and is noted to occur in some form in nearly all young children with AS [15, 60]. Hyperactivity/excitability occurs more often in AS than in other intellectual/developmental disability groups [61].

Frequently observed hypermotoric behaviors include restlessness, easy distractibility, inability to sit still, excessive activity, exuberance, and hyperkinetic or hyperactive movements [50]. Repeated movements of the hands, body, head, or face are also described as part of commonly observed hyperactivity [61]. Hyperactive behaviors decrease with age, with fewer adults being described as having hypermotoric behaviors than their younger counterpart [12]. Although it is unclear at what point hyperactive behaviors start to decrease, one study found that children under the age of 16 were reported to have significantly more hyperactivity than individuals over the age of 16 [60]. Whereas other cognitive and behavioral features are more common in individuals with a deletion, individuals with UPD were rated as having more hyperactivity than those with a deletion or a *UBE3A* mutation [13].

### Autism/autistic behaviors

Chromosome 15 and *UBE3A* have been found to be relevant for autism, increasing interest in autism traits within AS [62]. Individuals with the larger type I deletions (which more commonly result in mutations in the homologous to the E6-AP carboxy terminus domain ligases) and those who are more severely impaired are most likely to meet criteria for an autism spectrum disorder (ASD) based on the Autism Diagnostic Observation Schedule (ADOS) algorithms [5, 62–66]. The prevalence of individuals meeting criteria for ASD was higher in an AS sample than in a sample of individuals with Cri-du-chat syndrome, which has a similar severity of phenotype [67]. Those with AS and comorbid ASD score lower on measures of language, adaptive behavior, and cognition, and have a slower rate of growth over time than those with AS without ASD [63].

While autism symptoms are considered a part of the behavioral phenotype of AS, there is significant debate as to whether individuals with AS truly have comorbid ASD. In several studies, up to 50% to 81% of individuals with AS met diagnostic criteria for autism [62–64]. It is difficult, however, to determine whether it is the very low cognitive functioning driving the ASD diagnosis or whether the ASD comorbidity results in more cognitive impairment. For example, the gold standard diagnostic measures for ASD—the ADOS and Autism Diagnostic Interview–Revised—have mental-age floors of 12 and 24 months, respectively; those with AS who also meet criteria for ASD have been reported to have an average mental age of 6 months [63]. This leads to questions regarding whether the lowest functioning individuals with AS can be appropriately assessed for ASD with these measures. These questions have led some experts to conclude that the rates of autism comorbidity are elevated due to severe cognitive and language impairments and motor stereotypies [50, 68, 69].

Further, despite the overlapping genetic and clinical features, individuals with AS often enjoy social interaction, more than those with other ID conditions [67], contrary to one of the core features of ASD. In a study directly comparing individuals with AS and comorbid ASD and those with ASD only, those with AS and ASD had significantly more response to social smiles, response to their name, response to the facial expressions to others, shared enjoyment, and fewer repetitive or stereotyped behaviors than those with ASD only [64]. In addition, individuals with AS have been reported to be more behaviorally flexible than individuals with ASD only or nonspecific ID [70]. While children with AS engage in frequent object, body, and head stereotypes [71, 72], they are less likely to engage in repetitive behaviors than children with other ID conditions, including those with ASD only [64, 71].

### Aggression/irritability

There are differences in reports of aggression that may be due to age and subtype as well as how aggressive behavior is defined. In a study comparing irritability scores on the Aberrant Behavior Checklist among children with AS, Prader-Willi syndrome, or Smith-Magenis syndrome, children with AS received the lowest scores on irritability across groups, suggesting they present with less aggression than those with the other conditions [73]. However, physical aggression has been reported in up to 73% of adolescents with AS based on a parent survey [74] and in 72% of adults based on the Challenging Behavior Questionnaire [74, 75]; other studies of individuals encompassing a larger age range have reported much lower prevalence rates [55, 73]. However, there is little published research to document this potential trend or to help understand why aggression may be more prevalent in older individuals. Grabbing, pinching, and biting are the most frequently reported aggressive behaviors [15]. Nondeletion groups are reported to have more aggression/irritability as measured by the Aberrant Behavior Checklist [13]. Some researchers have argued that observed “aggressive” behaviors occur without malicious intent but instead as a method of social engagement [75], communication method [76], or for sensory stimulation [77].

### Self-injurious/repetitive behaviors

Self-injurious behaviors, as measured by the Repetitive Behavior Questionnaire, are not as common in AS in other ID conditions [74]. However, one study did observe that 52% of adults engaged in self-injurious behaviors [75], suggesting that these behaviors may be more common in older individuals. More research is needed to confirm this finding.

### Mouthing/feeding behavior

Chewing/Mouthing objects, eating nonfood items (pica), foraging food, and being a fussy eater are more common in AS than in individuals with similar severity of ID [51, 61]. Hyperphagic behaviors have also been described in children with UPD [78], although mouthing behaviors are more common among those with a deletion than in nondeletion subtypes [51]. Anecdotal reports suggest that these food behaviors may be more prevalent than has been previously reported and may be related to genetic overlap with Prader-Willi syndrome [79]. More research is needed to address the prevalence and severity of food-related concerns.

### Anxiety

Anxiety is not well studied in this population, but is thought to be under-recognized [12, 75], especially in adults. This is likely due to a manifestation of anxiety that is different from what might be expected—with behaviors being attributed to neurologic issues, pain, or gastrointestinal signs [16]. Individuals with AS may be sensitive to changes in routine or separation from a preferred caregiver, and many challenging behaviors may be a result of anxiety around these changes [12].

### Treatments

Studies exploring the use of functional analysis of behavior in children with AS suggest that escape, tangible obtainment, and/or social engagement seeking are the primary functions of challenging behaviors [77, 80]. One study found that individually provided functional communication training decreased behaviors significantly in 3 children [80]. Another study found that a year of intensive applied behavior analysis did not make a significant difference in adaptive behaviors relative to controls; however, trends in some developmental domains suggested that applied behavior analysis could be a promising intervention [81].

Several case reports have described behavior-based interventions that have shown some value for individuals with AS. A study examining the effects of discrimination training to help children with AS modify their social approach behaviors yielded positive results for all 4 participants trained. These children were trained to recognize an environmental cue as a sign of adult availability, reducing attention-seeking behaviors. These results have implications for reducing challenging behaviors related to social engagement [82].

Although some medications have been suggested as possibly being helpful to treat specific behavioral issues (e.g., stimulants for hyperactivity, antipsychotics for aggression) [16, 63, 75], no evidence-based studies have indicated the efficacy of these treatments in AS. Risperidone and methylphenidate have been prescribed for the management of hyperactivity in patients with AS, but both have limited benefit and side effects that include weight gain and lethargy [83]. (See Table 4 for a summary of treatment studies addressing behavior.)

#### Sleep

Sleep disturbances are considered part of the clinical diagnostic criteria for AS [9], although prevalence rates vary in the literature with approximately 20% to 90% of individuals with AS being described as experiencing sleep challenges [9]. Differences in reported prevalence are partially due to variability in measurement [84] (parent report vs. actigraphy) and the age of the individuals being studied. (See Table 3 for a summary of the natural history of sleep issues in AS.) Sleep disturbances are more common among younger children with AS, with 2 to 9 years being the most common age range for peak problems [84–86]. Some reports suggest that sleep problems improve with age [12], but other studies suggest that, in a significant percentage of individuals with AS, sleep problems continue into adolescence and adulthood [75, 85–87]. Unlike other aspects of the AS phenotype, there does not appear to be a difference in prevalence of sleep challenges among genetic subtypes [6, 7, 87]. Sleep issues are significantly more common in patients with AS than in individuals without AS but with similar cognitive impairments [16].

The most common type of sleep problem is insomnia with 35% to 60% of individuals with AS being described as having difficulty initiating sleep and/or maintaining sleep [61, 85, 87] and reduced total sleep time [88]. Night wakings and/or early wakings are typically reported more frequently than difficulties settling [86]. These awakenings occur multiple times per night and may be accompanied by other behaviors such as screaming [16]. Other frequently reported behaviors include snoring, enuresis, bruxism, sleep terrors, somnambulism, nocturnal hyperkinesia, and nocturnal laughing [85, 86, 89, 90]. Sleep breathing issues and periodic leg movements have been reported and are likely more associated with seizure disorder than cognitive impairment [89]. The sleep issues reported likely compromise quantity of sleep. On average, individuals with AS sleep between 5 and 6 h at night, although this is not always continuous hours of sleep and there is significant variability in sleep duration across samples of children [15]. Children with AS typically do not have daytime sleepiness even with poor nighttime sleep [85], suggesting that children with AS may need less sleep than same-age typical peers [61, 85]. Quality of sleep is also problematic, with reduced sleep efficiency and a higher percentage of slow wave sleep and subsequently less and shorter duration of rapid eye movement sleep found via sleep polygraph in children with AS compared with controls [88, 89].

Dysregulation of gamma-aminobutyric acid–mediated inhibitory influences on thalamocortical interactions are the likely cause of most sleep problems in AS [84]. Seizure disorder may exacerbate sleep disturbances [84, 90], as may other pathophysiological processes common in AS and not directly related to sleep, such as gastrointestinal discomfort and use of medications for seizures or behaviors [91]. Another study suggests that the *UBE3A* gene may be a novel genetic regulator of sleep homeostasis and that dysregulation of the sleep drive may be a key underlying variable in sleep problems in those with AS [92].

### Treatment

Individuals with AS may have significantly low levels of melatonin with a delayed melatonin peak [93], which may explain why melatonin has been a successful treatment option for some individuals [93–96]. Indeed, melatonin (0.3–5.0 mg) is one of the most commonly used sleep aids, and 5 separate case studies have documented its efficacy with children with AS [93–97]. The greatest support for melatonin as an effective treatment for sleep issues in AS comes from a placebo-controlled study in which 6 out of 8 children with AS were found to have decreased latency of sleep, decreases in nighttime awakenings, and increased total sleep time when treated with melatonin [94] as opposed to placebo. Treatment of central sleep apnea via sustained-release melatonin improved sleep rounds and reduced insomnia in a case study of a 9-year-old with AS [98]. Although melatonin is a promising treatment, it is not considered standard of care for everyone, primarily because it is ineffective for some individuals. The frequency and types of seizures may influence the efficacy of melatonin in some individuals [95].

A limited number of observational studies have evaluated medications used in the treatment of sleep problems in the AS population, although sleep medications, such as melatonin and Dipiperon, were reported by caregivers to be effective in 25% of cases [86, 87]. Some seizure medications, such as valproate acid and/or benzodiazepines, have also been described to aid in sleep [99].

Other approaches include behavioral therapy with or without medication. Improving sleep hygiene, reinforcing behaviors, and regulating sleep according to the sleep-wake rhythm have been shown to be effective ways to improve sleep onset and/or duration [97, 100]. (See Table 4 for a summary of treatment studies addressing sleep).

### Impact

#### Impact on the individual

Because of the severe cognitive and communication limitations inherent in AS, there are no studies that directly address the HR-QOL or lived experiences of individuals with AS. As such, in order to understand the likely impact of AS on the individual, we considered the outcomes of AS on the individual’s ability to act independently through mastery of functional skills, socialization with others, and their comorbid health risks.

### Functional skills

In studies exploring functional behavior in individuals across the age span (1–33 years) most report that individuals with AS do not achieve skills that are more advanced than what would be expected for a 3-year-old child [101, 102]. Daily activities require supervision and assistance, with individuals with AS requiring care throughout their lives [12, 103]. Self-help skills vary, although globally they are low and generally commensurate with an individual’s estimated mental age or cognitive functioning. Most individuals learn to walk unassisted (although many have an abnormal gait or minimal endurance for walking more than short distances), most can express likes and dislikes, and many can undress and feed themselves (although often only with a spoon) [8]. Help is usually needed for bathing, dressing, and food preparation [4]. Full toilet training is obtained for approximately 30% of individuals with AS, with most continuing to need pull-ups at night [8]. Voiding dysfunctions are likely contributors to challenges with toilet training [104]. Rates of incontinence are lower for AS than for a comparison group of individuals with comparably severe IDs [105].

Compared with other ID conditions (Down syndrome, Williams syndrome, Prader-Willi syndrome, fragile X syndrome), individuals with AS score significantly lower on all domains of adaptive behavior, as measured by the Vineland Adaptive Behavior Scales, Second Edition (VABS-II) [101]. For all other ID conditions in this study, adaptive behaviors were found to increase with age; however, this was not the case for AS. This was noted to be expected given the severity of cognitive impairment. Though all individuals with AS demonstrate significant impairments in all areas of adaptive behavior, individuals with a deletion tend to score lower than nondeletion subtypes.

Significant challenges with motor skills, including ataxic gait, tremors, and hypertonicity, may prevent individuals with AS from obtaining functional skills they may otherwise be able to obtain cognitively. Furthermore, while not life-threatening, NEM episodes are debilitating and can result in loss of previously obtained self-help skills, including feeding and independent mobility, as well as result in increased injuries due to falls. This very likely contributes to increased frustration and a reduction in quality of life for individuals with AS.

### Socialization

Socialization scores on the VABS-II have been reported to be higher than for other adaptive behavior domains [13, 24], which may reflect increased social interest. While this increased socialization may be a protective factor, there is some anecdotal evidence that, for some individuals, anxiety around separation from a preferred caregiver becomes more significant as they get older. In addition, as noted above, communication skills are significantly impaired for individuals with AS. As a result, many experience significant frustration in not being able to communicate, which is presumed to result in increased behavior challenges [39].

### Comorbid health risks

The most common health risk is seizures, which are almost universal (80%–95%) [16]. While most are well managed by medications, some patients require additional medical intervention (e.g., vagal nerve stimulation) or intensive home interventions (ketogenic diets), and some have retractable seizures that may ultimately lead to early death [3]. Gastrointestinal conditions in late-adolescents and adults (reflux, rumination, constipation, foreign body ingestion, and obesity) are also common and likely contribute to increased physical pain and reduced HR-QOL [75]. Finally, individuals with AS may be at higher risk for accidental injuries or early death due to poor motor planning or upper airway obstruction [106, 107], and the combination of fascination with water, motor impairments, and seizures have been fatal for some children [106].

#### Impact on caregivers

Given the severity of the AS phenotype, the burden on caregivers is thought to be high. However, there have been very few systematic explorations of burden on parents. Nothing has been published regarding the relative impact of caring for an individual with AS on the caregiver’s family planning, financial status, or work productivity.

Caregivers report high levels of fatigue, adverse effects on their social life, increased arguments with spouses or partners, and increased irritability, especially toward their family member with AS [86]. This is especially true for those whose family members with AS have more sleep problems. Sleep problems in individuals with AS are one of the most frequently reported stressors for families, affecting 65% of caregivers [108, 109]. Parents of children with AS who have sleep disturbances sleep less themselves and report high rates of stress [108, 109]. The less children slept and the more they woke at night, the more likely parents were to report negative mood and lower overall health for their children. Further, in a study of adults with AS, more than half (55%) of caregivers reported back pain or other chronic pain symptoms, and 48% felt moderate or severe anxiety about the future [75].

Another study found that parents of children with AS report more stress than parents of children with Prader-Willi syndrome and that parenting stress for both conditions was related to the children’s behavior challenges [110]. Parent stress was reported to be higher in parents of children with imprinting defects and UPD than for those with a deletion, perhaps because children with the deletion are typically lower functioning and, therefore, parents may have lower expectations for them relative to the higher-functioning children with ID or UPD [109]. One report noted that parents felt day-to-day management was easier for adults because there was a reduction in hyperactivity [12].

Reports of deaths as a result of poor motor planning and/or upper airway obstruction [106, 107] are almost certainly a stressor for families. Parents report being generally satisfied with their child’s schooling, but report some concerns regarding communication with the school and district regarding level of services provided to their children [111].

One study explored experiences of typically developing siblings of children with AS; these siblings reported experiencing more sibling rivalry (feeling their parents paid more attention to the child with AS than them), and perceived themselves as being more dominant and nurturing in their relationship with their sibling with AS (compared with children with only typically developing siblings) [112]. However, siblings of children with AS did not report experiencing greater conflicts despite their sibling with AS displaying high levels of challenging behaviors.

### Economic burden

No studies exploring the financial or societal burden of AS were found in the review, although the lifelong and complex medical needs of AS are thought to result in significant economic burden.

## Discussion

Although AS is a rare disorder, the clinical features are severe and lifelong, resulting in significant individual and family burden, and presumably economic and societal burden. Several studies in Europe and elsewhere suggest rates around 1:15,000, however there are wide descrepancies in reported epidemeiology and no large population based studies. Low prevalence rates and awareness of AS cause many families to endure a lengthy diagnostic odyssey, with symptoms reported as early as 6 months of age, but the average age of diagnosis not being until 1–4 years later. Our review highlights that once diagnosed, the lack of standardized treatment protocols or approved therapies, combined with the severity of the condition, results in high unmet clinical needs in the areas of motor functioning, communication, behavior, and sleep. Despite the paucity of data on HR-QOL, the impact of AS on the individual and families/caregiver is thought to be significant given that most individuals with AS never obtain skills that would allow them to manage daily living tasks independently. As a result, many individuals require significant lifelong support just to get through their day; for nearly half of the individuals with AS, this includes toileting and feeding.

Significant motor challenges further limit functional skills, and epileptic seizures and NEM can make these limitations worse as the individual gets older, often leading to loss of previously obtained skills. There is a scarcity of data on how specific motor impairments affect HR-QOL beyond functional ability and independence of individuals with AS. There is also a lack of consensus on which assessment(s) are recommended to measure change in the various aspects of motor skills in those diagnosed with AS.

Sleep is significantly disrupted in individuals diagnosed with AS, which contributes to caregiver fatigue and stress, and likely impacts their physical and mental health. Sleep disturbances in AS are multifactorial; the impact of aberrant gamma-aminobutyric acid transmission, cognitive impairment, hyperactivity, concomitant medications, sleep hygiene, and a comorbid seizure disorder on sleep regulation are mostly unknown.

All of these issues are likely exacerbated by lack of verbal communication in the individual with AS. Questions remain regarding the capacity for individuals with AS to communicate more effectively given the significant cognitive and motor impairments inherent in AS. Although many individuals with AS find ways to communicate some of their needs and wants through gestural communication, for others their inability to express themselves may result in frustration leading to increases in aggressive or maladaptive behaviors and is likely stressful for parents or caregivers trying to determine the needs of the individual. There is also a lack of information about the impact of communication impairment on the overall HR-QOL of the individual with AS and their families.

Although many individuals with AS are socially interested and smile and laugh often, these generally more positive traits can be overshadowed by hyperexcitability, anxiety and aggression—including biting and grabbing. Although these behaviors are reportedly the most problematic for individuals with AS and their caregivers, there is very little literature describing the prevalence, severity, and nature of aggressive behaviors, self-injurious behaviors, and anxiety in individuals diagnosed with AS. The majority of these features are chronic, and those behaviors that do decrease with age (e.g., hyperactivity) may be replaced by ones that are potentially more stressful (e.g., aggression and anxiety). Clinicians and researchers use various instruments to measure change in these behaviors, but there is little consensus on which are the most sensitive, reliable, and valid to measure change over time. Furthermore, there are no data that map the severity of behavioral symptoms or disease severity to functional ability or level of independence, caregiver burden, economic burden, or costs to families and payers.

Unfortunately, there are no approved treatments for AS, no current treatments that address the underlying etiology of AS, and no clear guidelines for symptom-based interventions in this population. Currently, treatments are symptomatic and largely limited to amelioration of seizures and reduction of sleep disturbances [113, 114], and there are few empirically based studies of behavioral interventions. While psychopharmacology has been suggested as possibly reducing behavior symptoms, this has been based primarily on anecdotal reports and very little is known regarding true efficacy and the safety profile of these medications in the AS population. Case studies suggest that intensive therapies such as physiotherapy, augmentative or alternative communication strategies, and functional behavioral analyses can make some difference for some individuals with AS.

As of this writing, all clinical trials that have attempted to improve outcomes have failed [91]. Clinical investigations that have been undertaken include those studying dietary supplements aimed at hypermethylating the maternal locus and trials of minocycline and levodopa, all of which showed some effect, but did not lead to significant improvements in neurodevelopment.

## Conclusion

AS is a severe, lifelong, rare genetic condition that results in significant functional limitations and likely poor HR-QOL for individuals and their caregivers. No standard of care or approved treatment currently exists for AS, and current treatments are symptomatic with limited utility, suggesting a high unmet clinical need. Given the likely high burden of AS, new treatments that target the etiology of the syndrome that result in even small improvements in features of the syndrome may be clinically and economically meaningful for patients and their families.

## Abbreviations

ADOS: Autism Diagnostic Observation Schedule
AS: Angelman syndrome
ASD: autism spectrum disorder
BP: breakpoint
HR-QOL: health-related quality of life
ID: intellectual disability
NEM: non-epileptic myoclonus
PRISMA: Preferred Reporting Items for Systematic Review and Meta-Analysis
SSRI: selective serotonin reuptake inhibitor
UBE3A: ubiquitin-protein ligase E3A
UPD: uniparental disomy
VABS-II: Vineland Adaptive Behavior Scales, Second Edition
